# Classification of Parkinson’s Disease Genotypes in *Drosophila* Using Spatiotemporal Profiling of Vision

**DOI:** 10.1038/srep16933

**Published:** 2015-11-24

**Authors:** Ryan J.H. West, Christopher J.H. Elliott, Alex R. Wade

**Affiliations:** 1 Department of Biology, The University of York, York; 2 Department of Psychology, The University of York, York

## Abstract

Electrophysiological studies indicate altered contrast processing in some Parkinson’s Disease (PD) patients. We recently demonstrated that vision is altered in *Drosophila* PD models and hypothesised that different types of genetic and idiopathic PD may affect dopaminergic visual signalling pathways differently. Here we asked whether visual responses in *Drosophila* could be used to identify PD mutations. To mimic a clinical setting a range of flies was used. Young flies from four control lines were compared to three early-onset PD mutations *(PINK1*, *DJ-1α* and *DJ-1β*), and to two other neurodegenerative mutations, one in the fly *LRRK2* orthologue (*dLRRK*) the other in *eggroll*, a model of general neurodegeneration in *Drosophila*. Stimuli were contrast reversing gratings spanning 64 spatiotemporal frequency combinations. We recorded the steady-state visually-evoked response amplitude across all combinations. We found that the pattern of neuronal responses differed between genotypes. Wild-type and early-onset PD flies formed separate clusters; the late-onset mutation is an outlier. Neuronal responses in early-onset PD flies were stronger than in wild-types. Multivariate pattern analysis grouped flies by PD/non-PD genotype with an accuracy >85%. We propose that machine learning algorithms may be useful in increasing the diagnostic specificity of human electrophysiological measurements in both animal models and PD patients.

Parkinson’s disease, first described in 1817 by James Parkinson^1^, is a chronic, progressive neurodegenerative disease affecting ~0.2–0.3% of the population, with an increased prevalence of ~1–2% in those aged over 50^2^,^3^. It is the second most common neurodegenerative disease, and is characterised by a progressive loss of dopaminergic neurons within the nigrostriatal pathway.

Whilst commonly considered a motor disorder, characterised by postural instability, bradykinesia, rigidity and tremor, a number of visual perturbations have also been reported in PD patients^4^,^5^,^6^. These include, but are not restricted to, hallucinations^7^, perturbed visual acuity^8^, double vision (Diplopia)^9^, dry eyes^10^, altered contrast sensitivity^11^, and light sensitivity^12^. Some of these symptoms, such as double vision, can be contributed to motor deficits and others (such as hallucinations) may result from dopaminergic medication. However, problems such as altered light and contrast sensitivity likely result from perturbed signalling within the visual system, as a result of dopaminergic neuron loss: It has been demonstrated that amacrine cells within the human retina, involved in light adaptation and the detection of the edges of shapes, are dopaminergic^13^,^14^ and that PD patients have reduced dopamine within these cells^15^.

While PD was once considered to have only a weak genetic component, the discovery of genes associated with PD (including *α-synuclein*, *parkin, PINK1, DJ-*1 and *LRRK2*) has revolutionised the study of the disease using genetically tractable model organisms, such as *Drosophila*^16^. Significant conservation of the cellular and molecular mechanisms regulating neuronal development and function within the CNS between flies and humans has established *Drosophila* as a highly successful model of PD. *Drosophila* researchers have elucidated the cellular function (and interactions) of many PD genes^17^,^18^,^19^,^20^ while recent advances have enabled us to begin to understand the associated neurophysiological changes^21^,^22^.

Although the superficial surface of the *Drosophila* eye bears little resemblance to that of vertebrates, the underlying retinal networks show many homologies. This was first recognised by Cajal, who pointed out that the fly amacrine cell and medulla intrinsic neurons are homologous to the vertebrate horizontal and amacrine cells, all mediating lateral interactions^23^. At the synaptic level, both fly and vertebrate synapses are dominated by multiple contact synapses^24^. Both visual systems project centrally, with fly and mammalian retinal neurons terminating in the medulla and lateral geniculate nucleus (LGN), respectively. Here there is a layered output, with both organisms separating colour, motion and other aspects of vision, into different layers of the nervous system^24^. Developmentally, many of the genes responsible for eye formation in flies have counterparts in mice and humans (e.g. *Pax6, eyeless*) and it is possible to identify structurally and functionally homologous counterparts in the visual systems of the two species^25^. Similarly, rapid progress is being made in understanding similar mechanisms of the specification of photoreceptor mosaics^26^. Such is the degree of similarity that there is little doubt that both systems have both a common neurogenetic ancestor and that both have also been constrained throughout evolutionary history to solve common problems in decoding the environment.

Previously we have used electroretinograms (ERG’s) and steady state visually evoked potential (SSVEP) recordings, in response to a full-field flash stimulus, to demonstrate perturbed visual function in *Drosophila* carrying PD related transgenes on the first day of adult life^27^. These same techniques indicate that older flies show profound loss of visual responses^21^,^28^. Our measurements demonstrate that mutations associated with PD have functional effects in the visual systems of these models at developmentally early stages. In this paper we ask whether a more complex measurement of visual function might allow us to discriminate between multiple PD genotypes, as well as between PD and control animals. To do this, we drew on fly stocks with mutations in genes whose human homologues are associated with early onset PD in humans: (*DJ-1α*, *DJ-1β* and *PINK1*). Although the precise mutations in these fly genes differ from those seen in human patients, they are, nevertheless, well-established models of human PD. We also examined a fly strain with a mutation in the fly orthologue of *LRRK2* (*dLrrk*), associated with *late* onset PD in humans. To compare our results from PD-related mutations to a non-PD disease phenotype, we also included a gene mutation associated with a generalised model of neurodegeneration in *Drosophila*, *eggroll^1^* 29. These lines were chosen because they all had white eyes and externally appeared identical. Flies have two *DJ-1* homologs, *DJ-1α* and *DJ-1β,* both of which are enriched in the adult head^30^,^31^, and the mutant alleles we chose (*DJ-1α*^Δ*72*^ and *DJ-1β*^Δ*93*^) are nulls^31^. We also use two null mutants of *PINK1* (*PINK1*^*B9* ^32 and *PINK1*^5^,^33^). *PINK1* is also expressed in the head. The final PD-related mutation is a loss-of-function mutation *dLRRK*^*Ex1*^ 34. Since all the PD-related fly stocks were in the *w*^*1118*^ background, three wildtype strains also carrying the null *w*^*1118*^ mutation, obtained from different sources, were used as controls. A second null in the *white* gene (*w*^*1*^) was used as an additional control. This allows us to address the variability in visual contrast perception between wild-type and PD-related genotypes, within each group, and between genes for early and late onset forms of PD.

Here we build upon our previous assay by further translating methods commonly used in the study of human vision to the fly. We now record SSVEPs in response to the modulation of spatial patterns at a variety of frequencies, adding a spatial component to our assay and providing an increase in complexity and sensitivity. Using this method we have measured spatiotemporal response profiles of the *Drosophila* visual system and, in doing so, demonstrated distinct profiles for wildtype flies and those with PD related mutations. In addition to this we show that we can use these distinct profiles to correctly classify randomly assigned genotypes.

## Methods

### *Drosophila* Stocks and Maintenance

Responses were measured from ten different *Drosophila* genotypes. We chose five ‘PD genotypes’–four with early-onset PD-related mutations and one with a loss-of-function mutation in the *Drosophila LRRK* (*dLrrk*) gene associated with late-onset PD. All the mutant strains had white eyes. Our control group consisted of four different wildtype ‘white eyed’ strains originating in different laboratories. As an additional control we tested a well characterised model of non-PD neurodegeneration, a mutation in the *eggroll* gene (*eggroll*^1^). *eggroll*^1^, *w*^*1118*^, *w*^*1*^, *PINK1*^5^ and *dLrrk*^*Ex1*^ fly stocks were obtained from the Bloomington *Drosophila* Stock Center (Indiana, USA). *PINK1*^*B9*^, *DJ-1α*^Δ*72*^ and *DJ-1β*^Δ*93*^ stocks were generously gifted by Dr Alex Whitworth (The University of Sheffield, UK). *w*^*Dahomey*^(*w*^*Dah*^) flies were a kind gift from Dr Susan Broughton (The University of Lancaster, UK)^35^. The second *w*^*1118*^ stock (here referred to as *w*^*T *ü^) was a kind gift from Dr Tobias Rasse (University of Tübingen). The *DJ-1α*^Δ*72*^,*DJ-1β*^Δ*93*^, *dLrrk*^*Ex1*^and *eggroll*^*1*^were tested as homozygotes. The *PINK1*^5^ and *PINK1*^*B9*^ were tested as hemizygotes as the gene is on the X-chromosome. All *D*. *melanogaster* lines were raised in a 12 hr:12 hr light:dark (LD) cycle at 25 °C on standard cornmeal-yeast-sucrose medium.

### Preparation of Flies for Assaying

**Figure 1:**
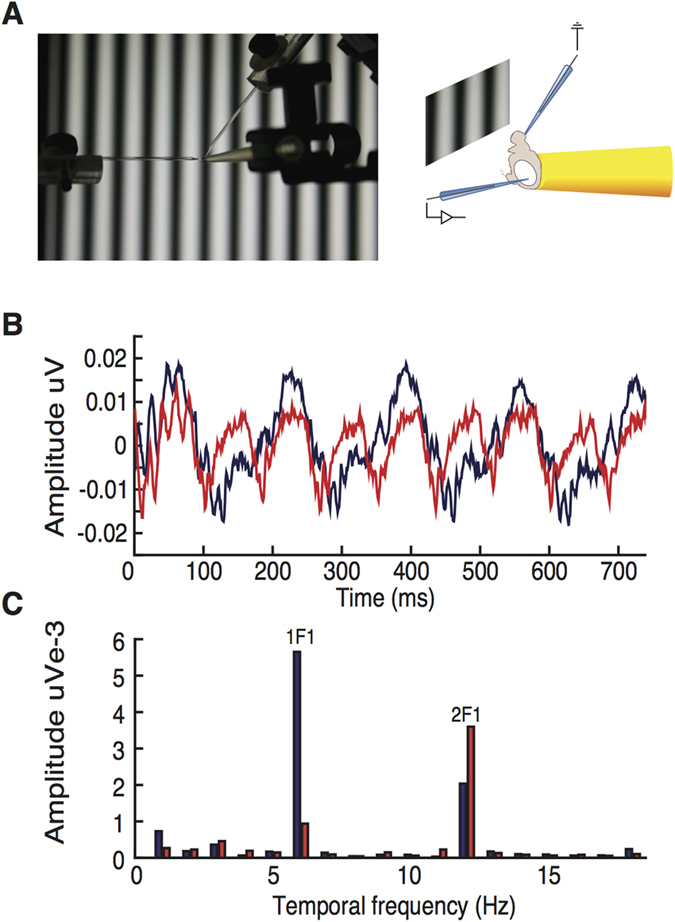
Data Acquisition and Analysis Pipeline for SSVEP (steady state visual evoked potential). (**A**)*Drosophila* are restrained in modified pipette tips and positioned in front of monitors displaying the contrast-reversing sine-wave grating stimuli. Recording and reference electrodes were placed onto the eye and into the mouthparts, respectively. (**B**) Examples of electroretinogram responses to 6 Hz contrast-reversing sine-wave stimuli of two different spatial frequencies (blue: .014 and red: .11 cycles per degree) in a ***DJ-1α***^**Δ*****72***^mutant. (**C)** Fourier transforms of the data in (**B)**. At low spatial frequencies, photoreceptor responses occur largely at the first harmonic while responses from deeper structures (LMCs, lamina, medulla) contribute to the second harmonic. At high spatial frequencies (red trace) the photoreceptors responses also shift to 2F.

Male flies were collected within 8 hours of eclosion and transferred onto standard yeast-sucrose-agar medium for 24 hours (12 hr:12 hr LD, 25 °C). After 24 hours, unanesthetised flies were aspirated into shortened pipette tips and restrained, with the head protruding, using nail varnish as described recently^27^,^36^ (Fig. 1A). Pipette tips holding the flies were mounted upon the apparatus, positioning the flies 0.22 m from the display monitor. Electrophysiological recordings were made using blunt glass pipette electrodes containing *Drosophila* simple saline (130 mM NaCl, 4.7 mM KCl, 1.9 mM CaCl_2_)^37^. Electrodes were placed gently into the mouthparts and onto the surface of the eye, for the reference and recording electrodes respectively (Fig. 1A).

### Stimuli

Stimuli were contrast-reversing sine-wave gratings at 98% contrast presented at a variety of spatial and temporal frequency combinations on a 144 Hz LCD monitor (XL2420T, BenQ, Taiwan). Stimuli were generated using the Psychophysics Toolbox^38^ on a Windows 7 PC and the monitor was gamma corrected for each LCD primary separately using a fibre optic photospectrometer (USB2000, Oceanoptics, Dumolin, FL). Temporal frequencies were chosen so that single reversal cycles comprised an integer number of monitor frames.

We tested all possible combinations of 8 temporal frequencies (1, 2, 4, 6, 8, 12, 18 and 36 Hz) and 8 spatial frequencies measured in ‘cycles per degree’ (0.014, 0.028, 0.056, 0.11, 0.22, 0.44, 0.88 and 1.76 cpd) in a random order. Each stimulus combination was presented for an 11 second trial during the randomized sequence with at least four seconds between each trial. The first one second ‘bin’ of each trial was discarded to eliminate onset transients and the remaining 10 bins were analysed using coherent (phase sensitive) frequency-domain average.

### Analysis

Contrast reversing grating patterns generate frequency- and phase-locked responses in the *Drosophila* VEP time series at integer multiples of the input frequency. When the spatial frequency of the stimulus is very low, most of the display will have a similar polarity at any one moment. In this case, the photoreceptor responses (the hyperpolarization and depolarization of the photoreceptors themselves) will track the reversal frequency ‘F’ while the responses from deeper structures such as the LMCs, lamina and medulla will occur at 2F because the neuronal transients generated by photoreceptor modulation occur for both on- and off- transitions^27^. However, at moderate to high spatial frequencies, the pooled photoreceptor responses also occur at twice the input frequency because different photoreceptors see different spatial locations and on average half the photoreceptors are looking at a positive-negative transition on each half of the reversal cycle (Fig. 1B,C). We restricted our analyses to the second harmonic (2F) of the input frequencies and computed the coherently averaged Fourier amplitude for each condition. In other words, for the 1, 2 and 4 Hz inputs we analyzed responses at 2, 4 and 8 Hz respectively.

**Figure 2:**
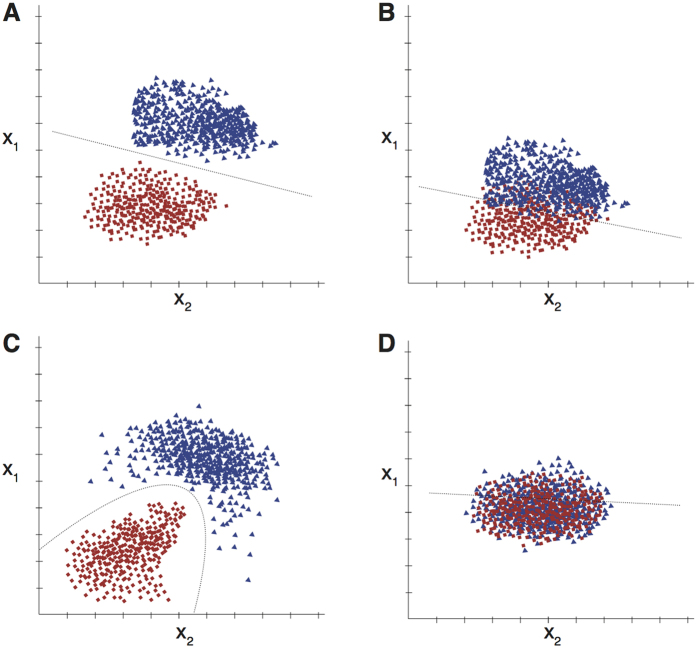
Discriminant Analysis. Using a machine learning discriminant analysis approach the ability to accurately classify datasets to a specific “class” can be assessed. In this example flies from two hypothetical genotypes “A” (blue triangles) and “B” (red squares) are separated into distinct “clusters” based upon the hypothetical variables x_1_ and x_2_ (perhaps representing maximum response amplitudes to stimuli presented at two different temporal frequencies). In example (**A)** the genotypes cluster at distinct points in dimensional space and so can be separated via a single linear boundary. Data obtained from a new “unknown” fly can be used to classify the genotype based upon which side of the classification boundary the response falls. In example (**B)** there are more subtle variations between genotypes creating a degree of overlap between clusters in the dimensional space and making classification more difficult. In example (**C)** we observe that the data are more easily separated using a non-linear boundary. In this instance pre-processing of the data to perform dimensionality-reduction could allow for a simpler linear classification boundary to be drawn. Finally in example (**D**) the genotypes cluster within the same dimensional space, making discriminant analysis difficult and the classification algorithm unlikely to perform above chance.

The fly visual system is highly sensitive to motion which can be decomposed into spatial frequency and temporal frequency components and tuning for both spatial and temporal frequency appears to be matched to environmental statistics in wild organisms^39^,^40^. While temporal frequency tuning to contrast reversing stimuli can be based on local inputs, tuning for spatial frequency requires long-range spatial computations that cannot be achieved at the level of individual photoreceptors or the large monopolar cells (LMCs). However, spatial frequency sensitivity can arise in deeper structures such as the medulla and lobular and elementary motion detectors (a fundamental part of the fly visual system) depend on long-range integrative mechanisms found in these locations^41^,^42^,^43^. Individual EMDs can respond to the spatiotemporal structure of our contrast-reversing gratings but because these stimuli contain no net motion, downstream integrative mechanisms will be largely silenced. Previous work from our lab indicates that our electrophysiological recordings can detect signals from deeper layers in the fly visual system (specifically, neurons in which two different frequency-tagged inputs are combined in a non-linear manner generating intermodulation terms). These signals are weak compared to the contributions from photoreceptors and initial synaptic transients but are statistically significant (see, e.g. Afsari *et al.*^27^ Fig. 2). We therefore sweep both temporal frequency and spatial frequency in our stimuli so that both superficial retinal layers and deeper structures can contribute to the classification performance.

Each randomized set of 64 trials was repeated in a different order 10 times for each fly resulting in a single recording session that lasted approximately one hour. Our recording rig ran two flies simultaneously to increase throughput and reduce inter-session variance. The data presented here are averages across all repetitions for each condition in individual flies. Within-fly averaging was performed coherently (i.e. by averaging complex frequency-domain data). In this type of data averaging, stimulus-evoked signals from different trials tend to combine additively because they have the same temporal phase. Conversely, non-stimulus-locked components tend to cancel to zero because, on average, the noise in different trials will have random phase. 20 flies of each genotype are represented in the classification datasets.

### Classification

We used a machine-learning discrimination analysis to assess our ability to assign flies to different genotypes based solely on their visual responses. Our SSVEP measurements provided 64 amplitude measurements per fly–one for each combination of spatial and temporal frequency. These 64 numbers can be thought of as locating each fly in a 64-dimensional feature space. If flies with similar genotypes have similar visual responses, then they will cluster together in this high-dimensional space. In addition, if flies with *different* genotypes have different visual responses, each genotype will form a separate cluster. The machine learning algorithm can then attempt to draw a boundary between clusters and use this to assign new datasets to a particular class.

This is illustrated in Fig. 2. Here, we imagine a simpler situation in which we measure two hypothetical variables (x_1_ and x_2_ – perhaps representing the maximum response amplitudes to gratings flickering at 2 Hz and 10 Hz) from flies of two different genotypes. It is clear that flies from genotype “A” (blue triangles) fall into a single cluster while flies of genotype “B” (red squares) fall into another cluster. Discriminant analysis will place a single, linear boundary between the two groups and, given a new measurement from an unidentified fly, we can assign it to a particular group with high accuracy by asking which side of the boundary it lies on.

In real life, the situation is not necessarily as simple as this. Firstly, the differences between the two groups might be very subtle–in our example this could manifest itself as an overlap of the two clusters (Fig. 2B) which, in turn, means that the overall accuracy of the classification is lower. We can still generate a linear boundary and assign any given ‘test’ data set to one class or another but there is an increased chance of a mis-classification because the boundary does not separate the two training sets cleanly.

Other factors that might compromise our classification accuracy include overfitting of the training data set (which will compromise our ability to generalize to new data) and the possibility that the boundary between the sets is non-linear (for example, in Fig. 2C the two classes are clearly separate in 2D space but the boundary between them is not a straight line). Mathematical pre-processing of the data (particularly dimensionality reduction) and more complex classification procedures can often be used to address overfitting and nonlinear boundaries but similarities between the responses from the target classes represents a fundamental and obvious limit on our ability to perform discrimination. Figure 2D illustrates an example of a situation in which even the best performing classification algorithm will perform at chance.

In the results section we show results from two slightly different classification procedures. In both cases we use Matlab’s (R1014a, Mathworks, MA) ‘classify’ algorithm to perform a simple linear discrimination between data in a high-dimensional space, as described above. In the first case, we perform classification on the raw data with no pre-processing. For each fly, all 64 data points contribute to the computation and no regularization is applied.

In the second case, we allow Matlab to perform regularization of the dataset before classification. Matlab’s regularization optimization procedure ‘cvshrink’ allows us to iterate over many possible combinations of a pair of variables (‘gamma’ – a smoothness’ constraint and ‘delta’: a noise threshold) to estimate the optimum number of components to include in the classification procedure (see also^44^). For the n by n pairwise classifications (Table 1) we applied this estimation for each pair of genotypes that we examined. For the n-way classification we applied this procedure once to the entire ensemble. Regularization always reduced the number of components used in the pattern classifier with the mean number of components or dimensions being 12 (min 9, max 24).

We note that the purpose of this paper is to demonstrate that discriminant analysis is a useful tool in the analysis of SSVEP datasets obtained from *Drosophila*. We are aware that more sophisticated pattern classification algorithms exist–for example, non-linear classifiers that permit the generation of curved boundaries in feature space or support vector machines (SVMs) that can map datasets into higher-dimensional spaces to optimize separation. However, as we show below, even simple linear classifiers perform well on our datasets and, in almost all cases, distinguish between flies of different genotypes successfully.

### Statistics

The accuracy of a machine learning algorithm can be assessed in several ways. One robust method is to perform a ‘leave one out’ analysis: training the classifier on data from all flies but one and then measuring its performance in assigning the remaining fly to a particular category. This can be repeated over all the flies in the dataset to obtain a score indicating the accuracy of the classifier for that particular dataset.

The ability of the classifier to generalize to other datasets can be assessed by Monte Carlo resampling methods: Training and test data can be synthesized repeatedly using random sampling with replacement from the original dataset. The performance of the classifier on each synthetic dataset is noted and the distribution of accuracies can be computed to provide an unbiased estimate of the mean score with confidence intervals^45^ or the probability of achieving the estimated performance level by chance (a ‘*p*’ value). In our results section we perform this bootstrapping using 10,000 iterations of the classification procedure drawing different samples from the same dataset with replacement and computing the ‘k fold loss’ of that sample each time. The distribution of performance estimates generated by this procedure is always unimodal and approximately normal. We consider classification to be statistically above chance if fewer than 1% of the bootstrapped 2-way classification probabilities are .5 or greater: the accuracy expected from a binary classifier operating at random. Similarly, we consider the 10-way classification performance to be significant if fewer than 1% of the bootstrapped classification trials have an accuracy less than .1 (1/10).

In Table 1 we show the results of this conservative classification performance estimate for each pair of genotypes that we measured.

## Results

### Spatiotemporal frequency maps

**Figure 3:**
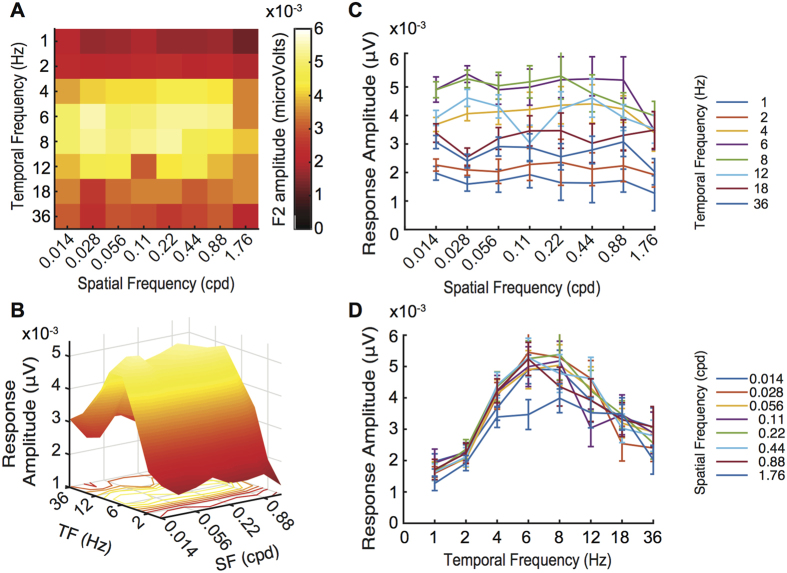
Spatiotemporal Response Profiles of a Wildtype Fly. (**A**)The two-dimensional heat map of the mean response amplitudes from a wildtype (*w*^*1118*^). (**B**) 3-Dimensional representation of the response profile and line plots collapsing across either space or time. (**C**) Mean Response at each Spatial Frequency. (**D)**. Mean Response at each Temporal Frequency. Error bars represent 1 SEM *N* = 20.

The data that we obtain from flies of a single (wild type) genotype (*w*^*1118*^) are illustrated in Fig. 3. The amplitude of the second harmonic at each combination of spatial and temporal frequency is presented in a colour coded heat map. High amplitudes are represented by ‘hot’ (bright) colours. A 3-dimensional surface rendering of the heat map is also shown in panel (B). The mean responses averaged across all temporal or spatial frequencies are plotted in panels (C) and (D) respectively.

Responses change as a function of both temporal and spatial frequencies. Responses tend to be relatively flat across low and intermediate spatial frequency but fall off sharply above 0.88 cycles per degree (cpd) (Fig. 3C). In comparison, the *w*^*1118*^ genotype shows a strong bandpass tuning for temporal frequency, peaking around 6 Hz (Fig. 3D).

**Figure 4:**
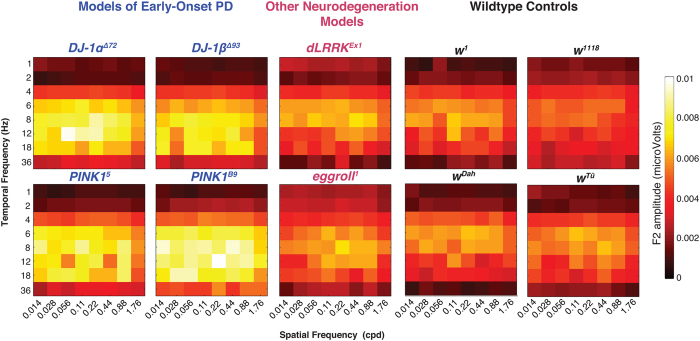
Early-onset Parkinson’s mutations elevate response across a range of spatiotemporal frequencies. Two-dimensional spatiotemporal profiles of all genotypes tested reveal distinct differences between wildtype and PD genotypes, as well as *within* each genotype. Mutations associated with early-onset Parkinson’s (*DJ-1α*^Δ*72*^, *DJ-1β*^Δ*93*^*, PINK1*^5^ and *PINK1*^*B9*^) show elevated response amplitudes at distinct regions of the spatiotemporal profile. Other mutations associated with neurodegeneration (*dLrrk*^*Ex1*^*, eggroll*^*1*^) show no elevation in response.

The location, amplitude and shape of this peak as well as more subtle features of the response profile change across genotypes. In Fig. 4 we show the spatiotemporal response profiles for all the genotypes that we tested. It is clear that there are significant differences both *between* the PD and wild-type groups and *within* each group. For example, all the *DJ-1* and *PINK1* flies show a much bigger response at low spatial frequencies than the wildtypes. In this paper, we are asking whether these changes contain enough statistical regularity to support a discrimination based on the responses from individual animals. To address this, we trained our classification algorithm on either pairs of datasets or the entire set of data and measured its performance in assigning a new dataset to the correct class.

### Two-way classification

**Figure 5:**
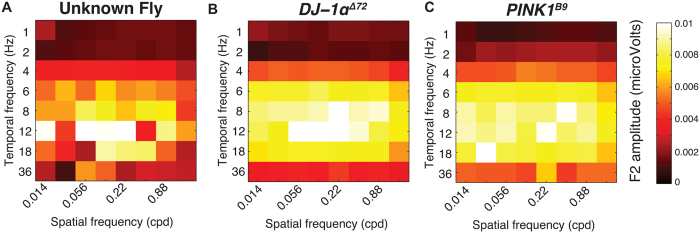
Flies of “Unknown” Genotypes can be Classified into Correct Genotypes Based on Spatiotemporal Profiles. (**A**) Fly of “unknown” genotype drawn from the *DJ-1α*^Δ*72*^ and *PINK1*^*B9*^ cohorts. (**B,C**) show the mean patterns of responses from two different early onset PD genotypes: *DJ-1α*^Δ*72*^ and *PINK1*^*B9*^. On average the response patterns in the two genotypes are clearly different. However, assigning any individual fly (for example, the one illustrated in (**A**) to a particular genotype is non-trivial. In this case, the classification algorithm correctly assigned the fly to the *DJ-1α* group. Once the datasets are regularized, the average accuracy is 88% with fewer than 1% of the resampled datasets giving an accuracy of less than 50%. In other words, we are relatively sure (p < .01) that a new fly of either genotype will be classified correctly. *N* = 20 (**A,B**). The full list of 2-way classification performances is provided in Table 1.

After supervised training on responses from these two genotypes, can our linear discriminator classify an ‘unknown’ fly correctly? Superficially, the averaged *DJ-1α*^Δ*72*^ and *PINK1*^*B9*^ heat maps (Fig. 5) are very similar, but the response peak in the *PINK1*^*B9*^ *is* sharper than that of the *DJ-1α*^Δ*72*^ genotype and shifted to slightly higher spatial frequencies. Figure 5A shows data from a single animal chosen at random from the ensemble of *DJ-1α*^Δ*72*^ and *PINK1*^*B9*^ recordings and the average response profiles from these two genotypes (Panels (B) and (C) respectively).

In this case, the classifier trained on other exemplars of these two classes correctly identified the animal as a *DJ-1α*^Δ*72*^ but for other pairs of response profiles drawn at random from the two genotypes, the classification might fail. In general, the accuracy of a classifier can be measured by its performance in a leave-one-out (LOO) analysis and its generalized performance can be estimated by bootstrapping this LOO analysis over many resampled iterations of the original dataset. The question we now ask is how accurately the classifier performs for each pair of genotypes, and specifically, how well will this performance generalise to new measurements of flies with unknown genotypes. We code performance on each pair as a mean percentage correct score (with a chance baseline of 50% in the 2-way classification) and we can estimate the probability that this performance would be above chance in a generalized population from the bootstrapping procedure. In the case of the *DJ-1α*^Δ*72*^ vs *PINK1*^*B9*^ data, the algorithm was able to assign a random fly to the correct category around 80% of the time using the raw, unprocessed response amplitudes. Optimizing the dataset through regularization improved performance to 88%.

In Table 1 we list the entire 10 × 10 array of two-way classification performance values: the expected classification accuracy for each pair of genotypes. Only scores with a bootstrapped significance of p < .01 are shown. For example, in the case where we train our classifier on regularized responses from *PINK1*^*B9*^and *w*^*1118*^flies, we expect to classify new flies with an accuracy of 86%.

Some genotypes are clearly more similar than others. For example, on average the *PINK1* flies show dramatically elevated response amplitudes compared to any of the controls (See Fig. 4) and are therefore classified at well above chance in these comparisons. On the other hand, different versions of the white eyed control phenotype are genetically and phenotypically more similar and it is therefore harder (although not impossible) to distinguish between them based on their visual response profiles. The most difficult classification is distinguishing (*w*^*1*^ and *w*^*Dah*^). Here, the classifier performs at only 67% accuracy. This is consistent with the observation that on average, spatiotemporal response profiles from these flies are extremely similar.

### PD vs non-PD classification

Given that we are, in general, able to classify flies successfully in cases where we use only two genotypes, might we be able to perform more general classifications–by assigning flies to larger phenotypic groups? These computations are likely to be more difficult than the two-way classifications shown in Table 1 because within-group variance is larger when multiple genotypes are assigned to a single class. However, they are relevant from the point of view of translating this classification system to a clinical environment where a single diagnosis (PD or non-PD) might be useful prior to more detailed genetic analysis. We therefore asked how well our simple linear classifier performed when presented with the entire dataset from nine genotypic classes. The baseline accuracy for this classification is 0.5 and, again, we performed bootstrapping to estimate the generalized performance over the entire cohort using a leave-one-out paradigm.

We found that even when PD and control genotypes were pooled, classification accuracy was better than 85% (p < .001). This is significantly greater than chance and suggests that responses from PD and control flies differ in some consistent manner. On inspection, the most significant difference would appear to be that the *DJ-1* and *PINK1* mutants show an increase in responsivity at intermediate spatiotemporal frequencies and a slight drop in responsivity at very low temporal frequencies.

### Classification across all genotypes

Next, we asked whether the classifier was able to assign individual flies to the correct genotype after training on the entire 10-class dataset. Performance is expected to drop on this type of task (because linear classification boundaries are more constrained by the presence of additional classes) but the chance classification rate is also lower (1/10 compared to 1/2).

**Figure 6:**
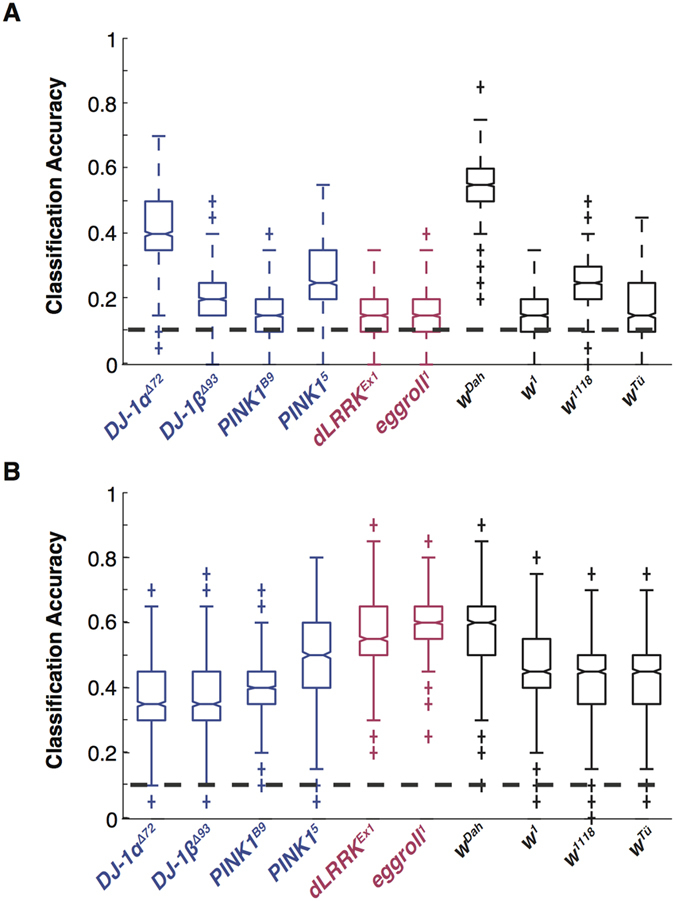
Spatiotemporal response profiles allow accurate “genotyping” of individual flies. (**A**)Following discriminant analysis machine learning of the entire 10-genotype dataset (*N* = 20 per genotype), individual flies were accurately classified into the correct genotypic “class” using the raw, un-processed data. All flies were classified correctly at a level significantly (p < .05) greater than chance (1/10, 0.1). (**B**) Regularizing the data significantly improved classification accuracies for all genotypes. All classifications were now greater than chance at p < .001 with the best performances approaching 60% accuracy.

Figure 6 shows the result of performing this analysis using either unregularized (6A) or regularized (6B) data (see methods). The data are plotted as box plots with the notches indicating the interquartile ranges. Surprisingly, even the unregularized datasets achieved a classification performance that was significantly above chance (p < .05, Fig. 6A). The *PINK1*^5^, *DJ-1α*^Δ*72*^, *w*^*1118*^ and *w*^*Dah*^ genotypes were most distinct with the classifier performing close to chance on the *w*^*1*^ genotypes.

When the data were regularized (Fig. 6B) performance increased markedly across the entire dataset. All phenotypes could now be classified at better than 30% accuracy and performance in some cases (*w*^*Dah*^, *dLrrk*^*EX1*^) approached 60%. In all cases the statistical significance of the performance measures was better than p < .001.

### Multidimensional scaling

Finally, we noted that the genotypes differ in how similar their average response patterns were. We therefore asked whether these response similarities could provide a direct readout of the genetic distance between different flies. The similarity between two response vectors can be conceptualized as a Euclidian distance in an n-dimensional space and it is trivial to compute the magnitude of all 10 × 10 pairwise distances in our dataset using a cross-correlation procedure. Once these distances have been computed, a multi-dimensional scaling algorithm^46^ can be applied to the data to visualize the clustering of different genotypes.

**Figure 7:**
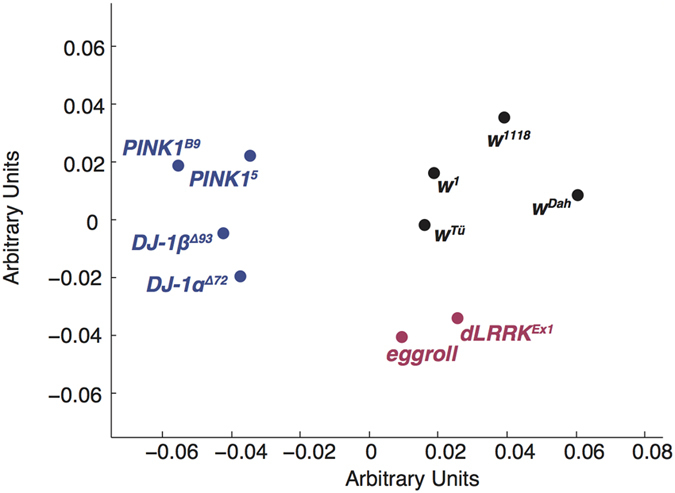
Multidimensional scaling output for mean response pattern similarity data. Euclidean distances between the average response vectors for each genotype in a high dimensional space were computed using cross correlation. These distances were then mapped to a two-dimensional space using multidimensional scaling (MDS). Genotypes of the same class (early-onset-associated, other neurodegenerative or control) generate distinct clusters. The orientation and scale of the axes are arbitrary.

This data visualization is shown in Fig. 7. A standard multidimensional scaling (MDS) procedure (Matlab’s ‘mdscale’) was applied to the 10 × 10 distance matrix computed from the mean, raw response vectors for each genotype. The first two dimensions of the MDS output are plotted with an arbitrary orientation and scaling. Control (*w*^*1118*^, *w*^*1*^, *w*^*Dah*^, *w*^*T*ü^), early-onset-associated PD (*DJ-1α*^Δ*72*^, *DJ-1β*^*Δ93*^, *PINK1*^*B9*^ and *PINK1*^5^), the late-onset-associated PD mutation (*dLRRK*^*Ex1*^) genotypes and general degenerative mutant *eggroll*^1^ are coloured as in Fig. 4. It can be observed that the control and early-onset-associated classes generate separate clusters, consistent with them having responses that are more similar to other members of the same class than to any other genotype in the set. The late-onset-associated genotype and eggroll are close together, outside any other group (their position lies outside the convex hulls of both the early-onset-associated and control flies) – consistent with the nature of their mutations being qualitatively different to those seen in the *DJ-1* and *PINK1* genotypes.

## Discussion

Here, for the first time, we have extended our SSVEP technique to spatially structured stimuli and show that subtle variations in the spatiotemporal response profiles measured from *Drosophila* PD models can be used to classify their genetic status. Once trained, the technique can distinguish between pairs of genotypes with accuracies ranging between 66% and 94% and can even classify flies drawn at random from the entire set of genotypes with an accuracy that is statistically better than chance. It is worth reiterating that all the animals used in this study were visually indistinguishable–both in their appearance and in their behaviour.

This method has considerable potential in the study of PD using animal models. Firstly, it allows us to identify critical sets of spatiotemporal frequency responses that distinguish early onset PD from control genotypes. This, in turn, provides a sensitive and accurate method for monitoring disease progression in these models. In flies, this might facilitate drug screening and the genetic dissection of molecular pathways implicated in disease pathology. It is interesting to note the ability to identify the subtle, yet significant, variance in visual responsivity seen between the white eyed control strains. In particular the *w*^*1118*^ and *w*^*Tü*^ flies, which can be distinguished between at around an 85% classification accuracy. Both of these strains are of an identical genetic background, *w*^*1118*^. However they have been maintained as separate stocks in different laboratories for the past decade. Any variation must therefore have arisen from spontaneous genetic events and we note another recent report of phenotypic divergence within populations of flies with relatively recent common ancestors^47^. Such variation is potentially comparable to distinct, globally separated, populations of humans as well as the endogenous genetic variation that one may observe in a clinical setting. In addition, the ability to classify between wildtypes, which show only subtle between-group variations, demonstrates the sensitivity of this assay.

Whilst the observation of elevated visual responsivity across the spatiotemporal range in all early-onset PD genotypes is of significant interest it may be unsurprising that all show a similar profile pattern. In fact it has previously been stated that “The clinical phenotype of *Parkin-*, *PINK1-*, and *DJ-1*-linked PD is indistinguishable”^48^. Despite this similarity, we show that this assay is sensitive enough to classify between different PD genotypes.

In previous work, we showed that the overall contrast sensitivity of the *Drosophila* visual system can be affected by the expression of a single PD-related human transgene^27^,^36^. Shortly after eclosion, flies expressing *hLRRK2-G2019S* in their dopaminergic neurons exhibit an increase in visual contrast sensitivity but visual responses in these same flies are markedly diminished in later life^28^. These data were obtained by measuring frequency-locked responses to modulations of a single wide-field light source (a high intensity LED) at a fixed alternation rate. We were therefore unable to test whether more significant (or different) changes in response sensitivity occurred at different spatial or temporal frequencies. After mapping the entire spatiotemporal response profile, it is clear that the spatial (0 cpd) and temporal (12 Hz) parameters used in the Afsari *et al.* and Mortiboys *et al.*, studies^27^,^28^ localize a portion of the response space that does exhibit higher amplitudes in young PD flies and that the *G2019S* results extend to other PD genotypes. However, it is also clear that a significant amount of extra information exists in other parts of the spatiotemporal parameter space.

Previous work in animal models^6^,^49^ has shown visual deficits associated with pharmacologically induced PD. These papers report reductions in response amplitudes or increases in response latency in older animals, consistent with the results from our previous work^28^,^36^. However, recent work in Drosophila^27^ and mice^50^ also identifies response amplitude *increases* in young animals with genetic forms of the disease that may correspond to pro-dromal biomarkers in humans.

Retinal dopamine levels are reduced in human PD patients^15^ and a wide range of human studies have also pointed to the possibility of changes in electroretinogram (ERG) or VEP (visual evoked potentials) latencies and amplitudes in PD patients^6^,^51^,^52^,^53^,^54^. These findings are somewhat heterogeneous: some groups find frank changes in VEP amplitude^6^,^55^ while others suggest that the abnormalities are most prominent in the phase of the VEP response, or are best observed using particular types of cone-isolating patterns^56^,^57^. However, prior to the discovery of PD-related genetic loci, the first being alpha synuclein in 1997^58^ and the most prevalent being *LRRK2* (2004)^59^,^60^, studies were unable to account for the different environmental and genetic origins of the disease. We now know that around 1–2% of sporadic and 2–5% of familial PD cases in European populations are caused by a single gain-of-function mutation in the *LRRK2* gene (*LRRK2-G2019S*)^61^. Several other distinct genetic forms of the disease have also been identified, including mutations in *PINK1, parkin* and *DJ-1*^62^,^63^,^64^. An outstanding challenge at this point is therefore to characterize potential VEP abnormalities associated with each human phenotype. In this paper we have focused solely on the response amplitude at different spatiotemporal frequencies but the statistical power of the technique may be improved if the phase, chromatic and achromatic contrast sensitivity, or masking properties of the responses were also considered. We are currently pursuing this approach in genotypically characterized human patients as well as animal models.

We are also investigating the neurophysiological origins of the variations that we observe as these may provide clues as to the function of the mutated genes and their relation to PD. One possibility is that dopamine release is impaired in the PD mutants. Fly photoreceptors have dopaminergic receptors, and the presence of dopamine slows their response to a 10 ms flash of light^65^. Dopaminergic terminals are also found in the lamina and medulla^36^,^66^,^67^and we would expect dopamine release in these areas to have a coordinated action. In the absence of dopamine, we would expect fly photoreceptors (and, we speculate) the lamina and medulla to respond faster and more strongly. The resulting hyperactivity increases the ionic flux across the cell membrane leads an increased demand for ATP. If the mitochondria are unable to maintain the supply of ATP, the production of reactive oxygen species will be elevated, and lead to an excitotoxic cascade (mitochondrial damage and mitophagy, autophagy, apoptosis).

Finally, the possibility of applying these machine learning approaches to a clinical environment is appealing but there is also considerable value in using *Drosophila* as animal models of PD. In particular, *Drosophila* represent attractive models for early drug testing: their genetics can be controlled tightly, they have a short generation time, they are exempt from ethical controls and drug administration is particularly straightforward. Our ability to monitor the effects of PD mutations and transgenes rapidly and accurately is a crucial part of this procedure. Once baselines have been established, these rapid assays will also allow us to detect subtle responses to therapeutic interventions.

## Additional Information

**How to cite this article**: West, R. J.H. *et al.* Classification of Parkinson′s Disease Genotypes in *Drosophila* Using Spatiotemporal Profiling of Vision. *Sci. Rep.* **5**, 16933; doi: 10.1038/srep16933 (2015).

## Figures and Tables

**Table 1:** Mean classification performances for pairwise classification analysis.

	*DJ-1α*^Δ*72*^	*DJ-1β*^Δ*93*^	*dLRRK*^*EX1*^	*PINK1*^*5*^	*PINK1*^*B9*^	*eggroll*^1^	*w*^*Dah*^	*w*^*1*^	*w*^*1118*^	*w*^*T*ü^
***DJ-1α***^**Δ*****72***^	–	**66**(65)	**87**(78)	**85**(80)	**88**(80)	**88**(80)	**92**(85)	**86**(84)	**89**(83)	**82**(76)
***DJ1-β***^***Δ93***^	–	–	**90**(87)	**70**(69)	**77**(69)	**86**(78)	**85**(86)	**80**(79)	**84**(79)	**78**(76)
***dLRRK***^***EX1***^	–	–	–	**93**(87)	**94**(93)	**89**(80)	**84**(76)	**87**(81)	**83**(76)	**74**(72)
***PINK1***^***5***^	–	–	–	–	**68**(68)	**90**(82)	**85**(83)	**72**(72)	**92**(89)	**88**(82)
***PINK1***^***B9***^	–	–	–	–	–	**89**(81)	**89**(91)	**85**(85)	**86**(86)	**89**(86)
***eggroll***^1^	–	–	–	–	–	–	**88**(83)	**81**(81)	**75**(75)	**82**(78)
***w***^***Dah***^	–	–	–	–	–	–	–	**67**(60)	**83**(81)	**75**(72)
***w***^***1***^	–	–	–	–	–	–	–	–	**80**(81)	**78**(78)
***w***^***1118***^	–	–	–	–	–	–	–	–	–	**82**(77)
***w***^***Tu***^	–	–	–	–	–	–	–	–	–	–

Regularized data in bold.
